# A methodology for identification and control of electro-mechanical actuators

**DOI:** 10.1016/j.mex.2015.04.001

**Published:** 2015-04-25

**Authors:** Tarek A. Tutunji, Ashraf Saleem

**Affiliations:** a Department of Mechatronics Engineering, Philadelphia University, Jordan; b Department of Electrical and Computer Engineering, Sultan Qaboos University, Oman

**Keywords:** System identification, Hardware-in-the-Loop, Electro-mechanical actuators, Mechatronics

## Abstract

Mechatronic systems are fully-integrated engineering systems that are composed of mechanical, electronic, and computer control sub-systems. These integrated systems use electro-mechanical actuators to cause the required motion. Therefore, the design of appropriate controllers for these actuators are an essential step in mechatronic system design. In this paper, a three-stage methodology for real-time identification and control of electro-mechanical actuator plants is presented, tested, and validated. First, identification models are constructed from experimental data to approximate the plants’ response. Second, the identified model is used in a simulation environment for the purpose of designing a suitable controller. Finally, the designed controller is applied and tested on the real plant through Hardware-in-the-Loop (HIL) environment. The described three-stage methodology provides the following practical contributions:

• Establishes an easy-to-follow methodology for controller design of electro-mechanical actuators.
• Combines off-line and on-line controller design for practical performance.
• Modifies the HIL concept by using physical plants with computer control (rather than virtual plants with physical controllers).

Simulated and experimental results for two case studies, induction motor and vehicle drive system, are presented in order to validate the proposed methodology. These results showed that electromechanical actuators can be identified and controlled using an easy-to-duplicate and flexible procedure.

## Graphical abstract

**Figure:**
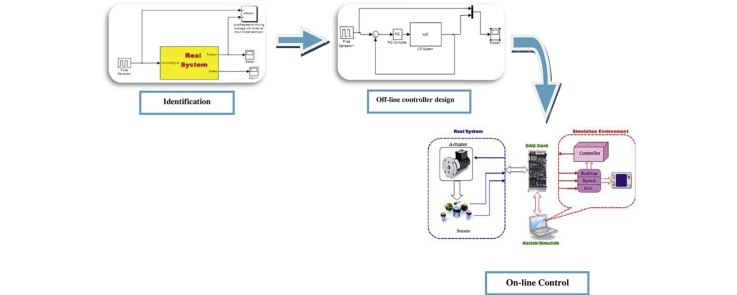

## Method details

**Fig. 1:**
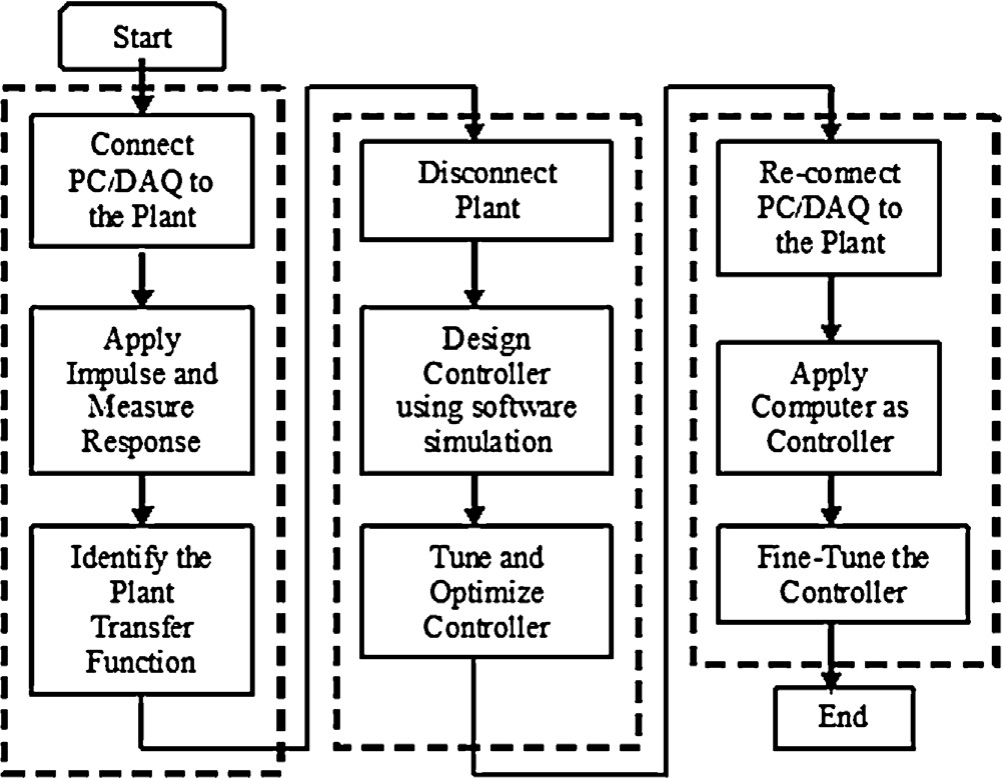
The proposed three-stage methodology.

The aim of the proposed methodology is to choose and apply appropriate controllers to general electro-mechanical actuators. It is assumed that the plant model is not known in advance. The idea is to approximate the plant model by identifying the transfer function using real-time data. This implies the use of computer acquisition arrangement connected directly to the plant. Then, the plant is disconnected and controllers are applied to the identified model in a simulation environment. This provides the flexibility of trying different control methods to the model without causing damage or downtime for plant. Once the controller is designed and tuned in a pure simulation environment, the plant is then re-connected to the computer acquisition arrangement where the computer is used as the controller. These steps are shown in Fig. 1. The advantages of the proposed methodology are: optimizing time resources and minimizing the cost as a result of online identification and offline controller design. The three steps are described in next.

### Stage one: identification

**Fig. 2:**
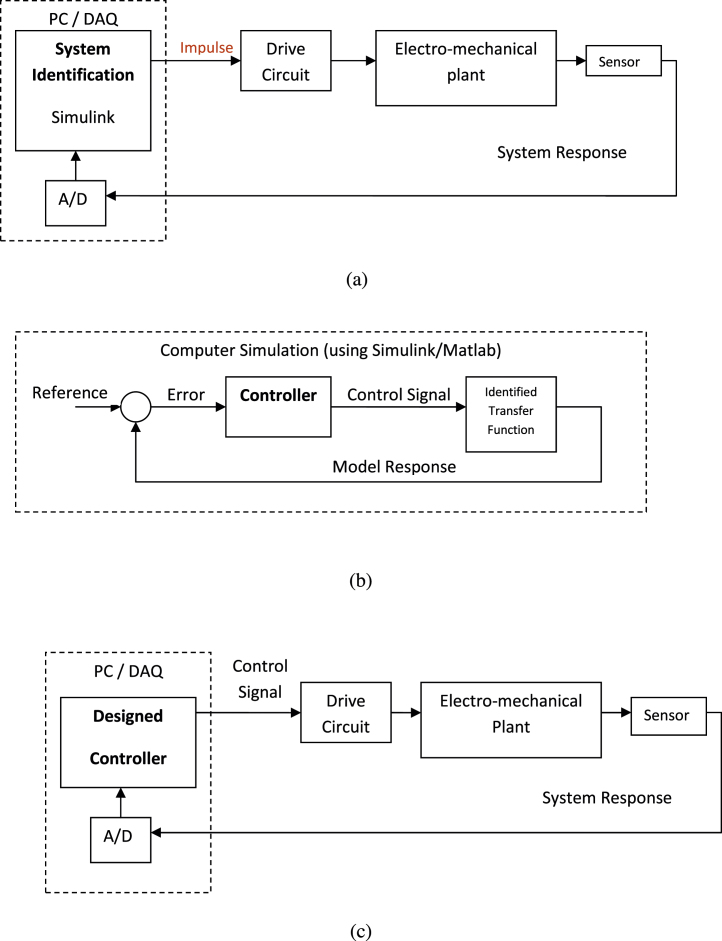
(a) Online identification, (b) off-line controller design, (c) online control.

Identification is the process of building a dynamical mathematical model using measured data in a real-time environment [1]. The mathematical model built is the estimated transfer function of the identified system. In this stage, the actuator is connected to the computer through data acquisition card. A computer program using Matlab/Simulink is used to apply an impulse signal to the plant as shown in Fig. 2a. This in turn, activates the plant. For implementation purposes a pulse was used instead of an impulse where the period of the pulse was short enough to represent an ideal impulse but long enough to activate the actuator to its desired settling speed. The system response is then acquired and recorded back to the computer. The computer software uses these input/output data patterns to build a model by minimizing the error, between the model and plant.

### Stage two: controller design

The identified transfer function in stage one is used to replace the plant behaviour in a simulation environment. Here, the plant is disconnected from the computer acquisition arrangement and can be taken back to its productive environment. At this stage, the appropriate controller structure is selected and the controller parameters are chosen. The controller design is performed in a pure simulation environment and therefore different controllers can be tested while damage to the plant is avoided and the downtime of the plant is minimized as shown in Fig. 2b. The controller design methods can vary from simple proportional-integral-derivative (PID) controllers [2] to intelligent neuro-fuzzy controllers. This process is referred to as Simulation-in-the-Loop (SIL), where a software version of the controller is tested with the simulated plant. The scope of this paper is not in the controller design development because the appropriate controller method varies among different systems. The scope here is to establish the three-stage procedure that can be applied to general dynamic systems.

### Stage three: online control

The designed controller in the previous stage is used as the actual controller for the plant. The plant is therefore reconnected to the computer in order to test the controller and carry out fine tuning online. This extra tuning might be necessary to capture any differences between the identified model and the real plant. In this study, the computer is used as the controller as shown in Fig. 2c. However, this procedure can be also extended to other controller options such as Programmable Logic Controller Digital Signal Processer. This stage is referred to as HIL control.

## Case studies

This section presents the results of applying the proposed methodology to two case studies: induction motor and vehicle drive system. The scope of this paper is concerned with the validation of the three-stage methodology described in the previous section rather than applying the best control method for the selected systems.

Traditionally, HIL environment is used to test real controllers with virtual plant models in order to validate the controller design. Such applications include unmanned air vehicles where the controller testing on the plant is expensive because of the high probability of failure. In the context of this work, HIL was utilized differently where virtual controllers were tested on real plants. Some researchers refer to this step as control prototyping [3].

The reason for using HIL in this context is to optimize time resources and minimize the cost: the system to be controlled will not be used during the experimentation of the controller design and therefore the plant downtime will be minimized. This might be a crucial time saving issue when the plant is used in production line. Equally important, damage to the plant due to inappropriate parameter values is avoided.

## Case study one: induction motor

**Fig. 3:**
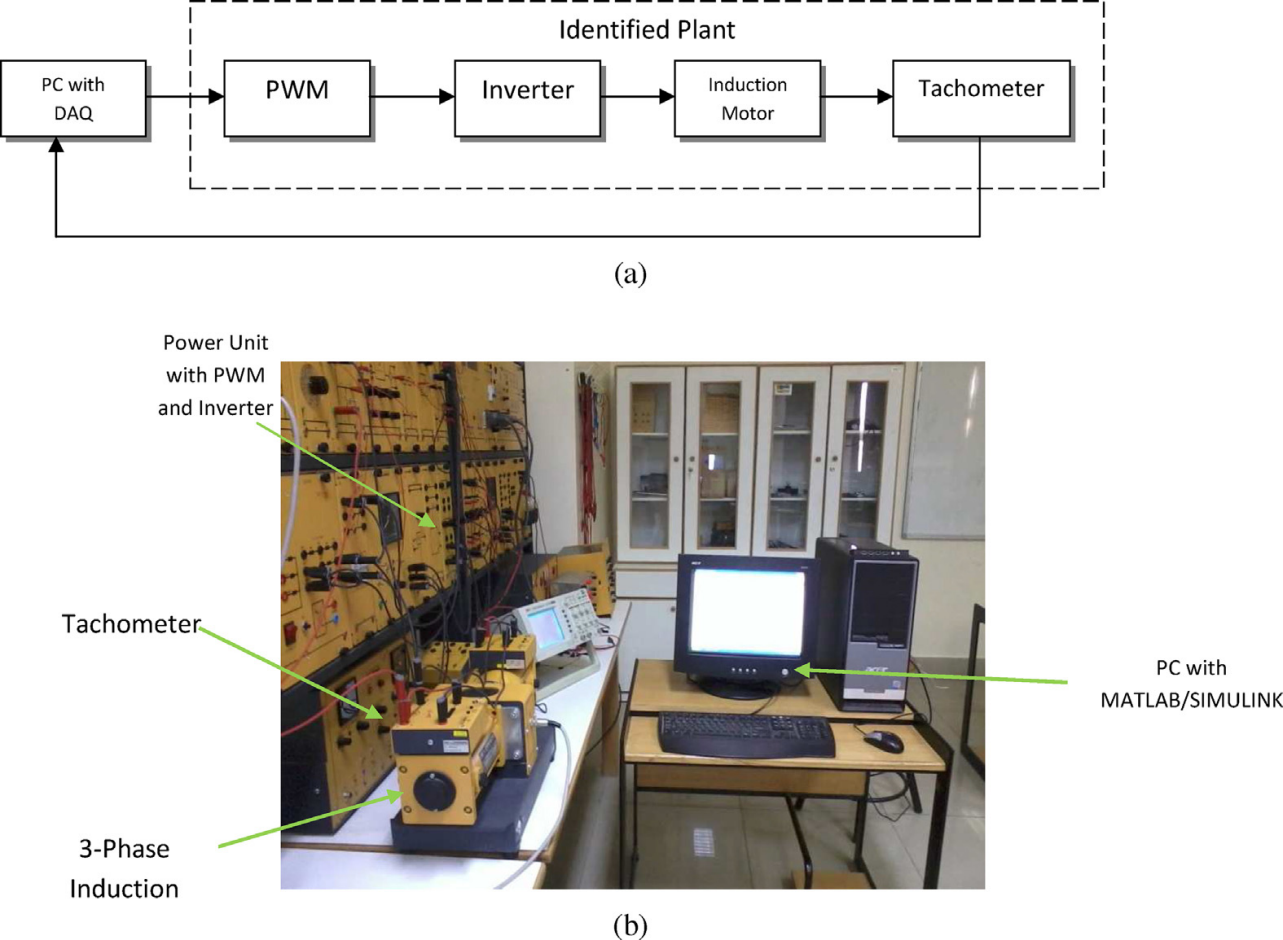
(a) Block diagram for experiment used, (b) induction motor experiment set-up.

A 3-phase squirrel cage induction motor was used in this study with specifications shown in Table 1. The block diagram of the test setup is shown in Fig. 3a. Note that the plant included pulse width modulator (PWM), inverter, and tachometer. The actual test rig in the laboratory is shown in Fig. 3b.

### Stage one: identification

**Fig. 4:**
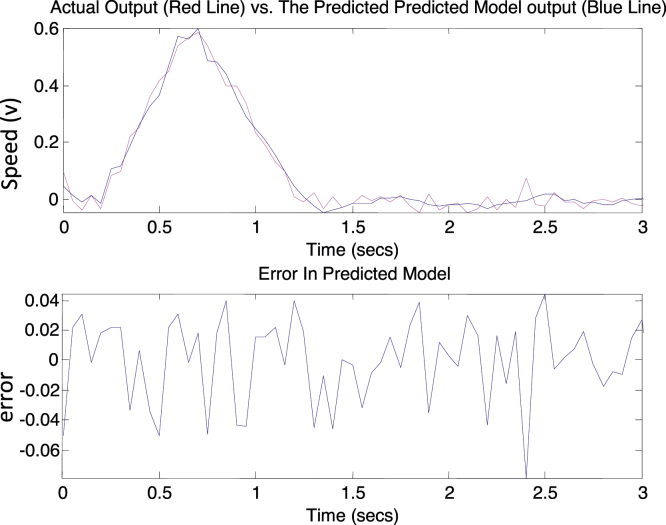
Plant response versus identified response.

A 5-volt pulse of 0.15 s duration was applied from the computer to the induction motor plant as the input while motor’s speed was the output measured by the tachometer and sent to the computer. The sampling rate used was 0.05 sample/s. These input–output data patterns were used in a recursive least square (RLS) algorithm to estimate the system transfer function. Fig. 4 compares the pulse response between the plant and a 6th order identified model where the output is the speed measured in volts (1 V ≈ 1000 RPM). The results show that the model was able to follow the original response with minimal error. The model order was selected based on several tries [4] and the identified transfer function was found to be:

$$H(z)=\frac{{0.1z}^{6}-{0.066z}^{5}-{0.2893z}^{4}+{0.4615z}^{3}+{0.2238z}^{2}+0.04004z}{z^{6}-{0.7z}^{5}-{0.6037z}^{4}+{0.06655z}^{3}+{0.3929z}^{2}-0.06332z+0.00384}$$

### Stage two: controller design

**Fig. 5:**
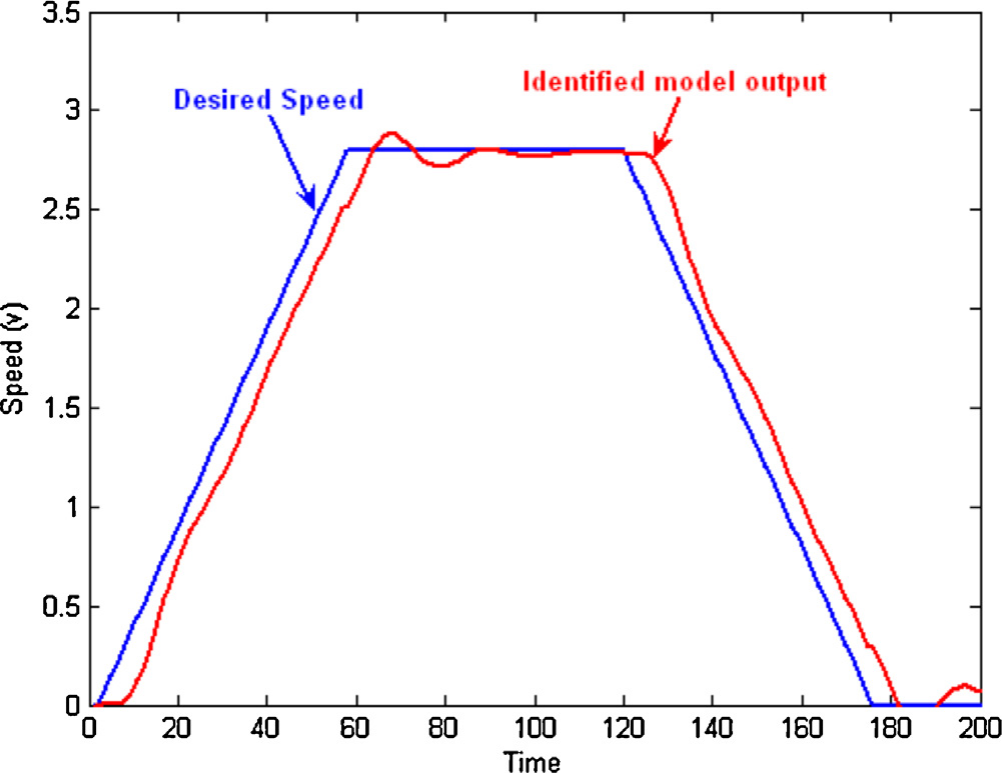
Identified model controlled response off-line with PID controller (*P *= 3.8, *I *= 0.002, *D *= 0.5).

Second, the approximated transfer function was used within Simulink in order to design an appropriate controller. Simulation was used for selecting the appropriate structure and parameter tuning. A PID controller was selected and the parameters were tuned to: *P *= 3.8, *I *= 0.002, and *D *= 0.5 was used. The controlled speed response of the identified model to a trapezoidal speed profile is shown in Fig. 5.

### Stage three: online control

**Fig. 6:**
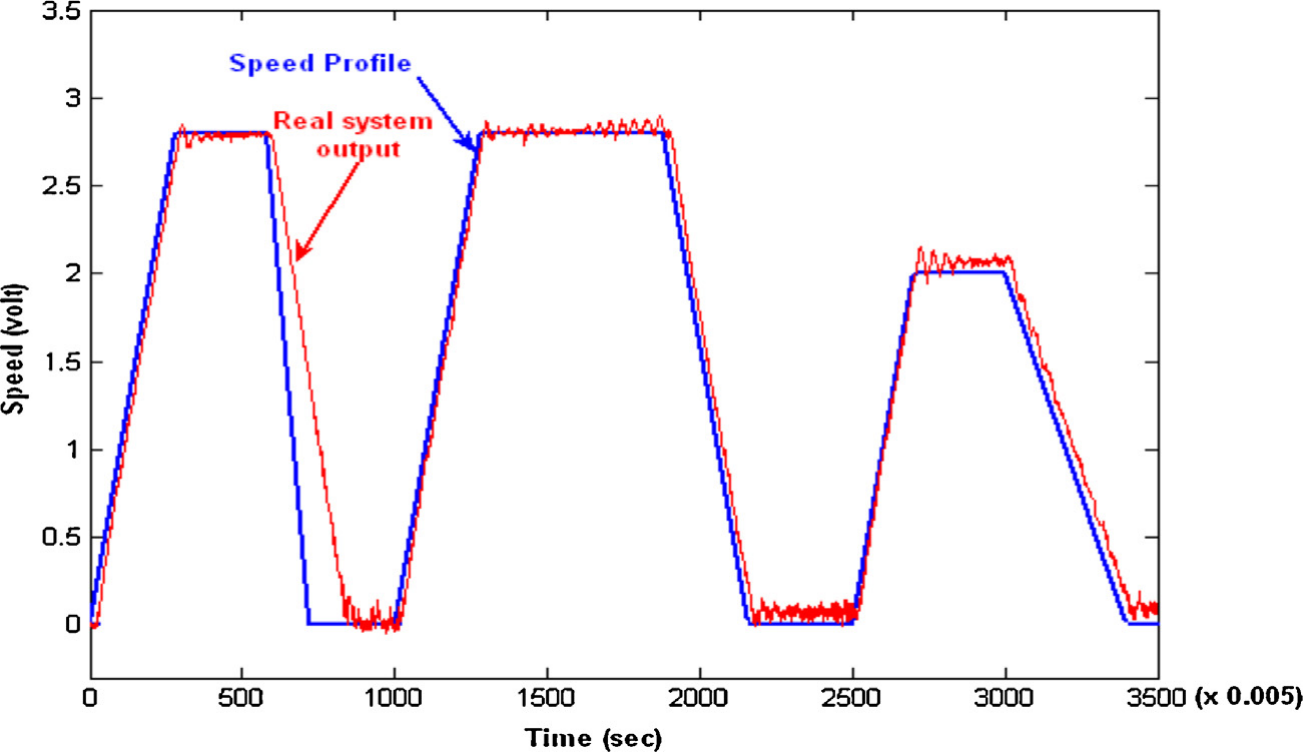
Response of the induction motor under test with PID controller.

The designed controller (with the same tuned parameters) was then applied from the computer to the induction motor system in HIL environment. The final results are shown in Fig. 6 where the controller succeeded in following the desired signal. These experimental results validated the identified model used in the controller design.

## Case study two: vehicle drive system

**Fig. 7:**
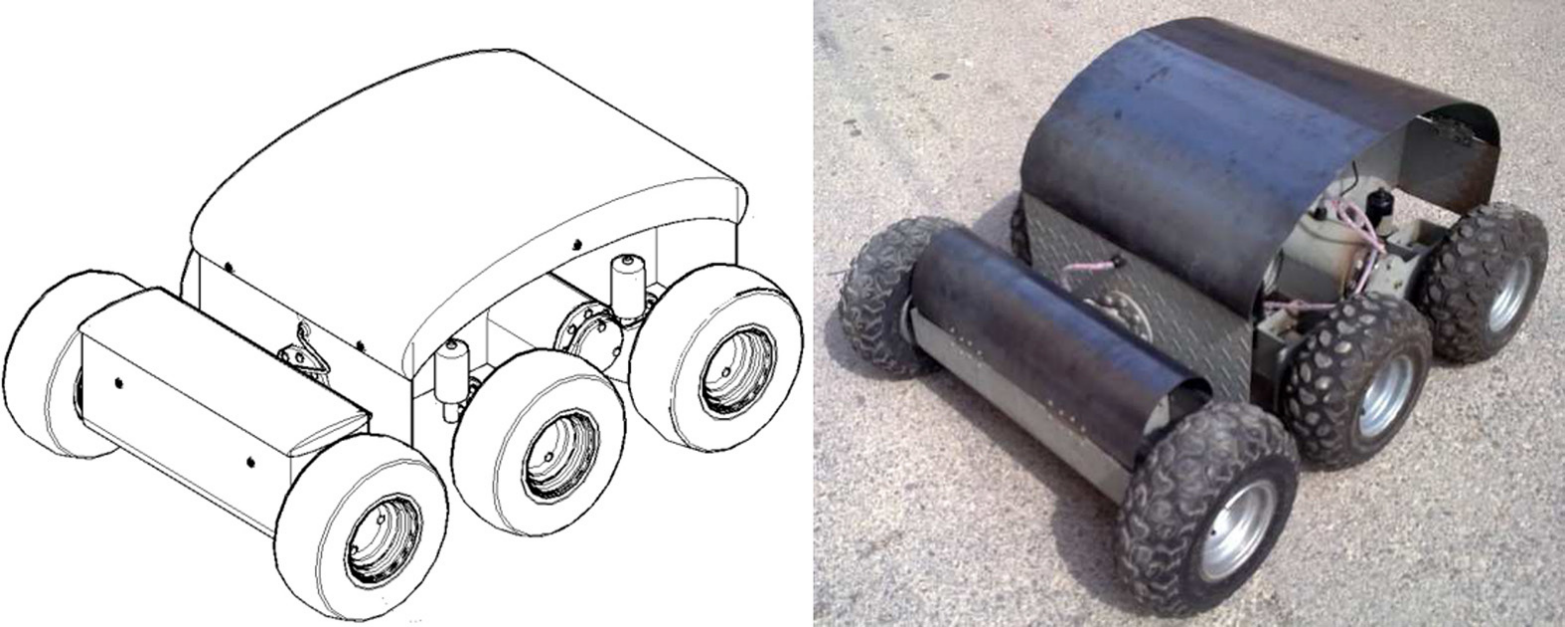
Developed UGV.

Unmanned ground vehicles (UGV) are robotic platforms that are used for both civilian and military applications to perform dull, dirty, and dangerous activities. One of the main challenges in developing such vehicles is the control system design. A UGV with six independent wheels was developed as depicted in Fig. 7. Each wheel had a driving motor which had to be controlled accurately by the main controller in order to perform accurate speed and steering for the vehicle. In this part, the design and implementation of the controller for the driving motors using the proposed methodology is presented.

**Fig. 8:**
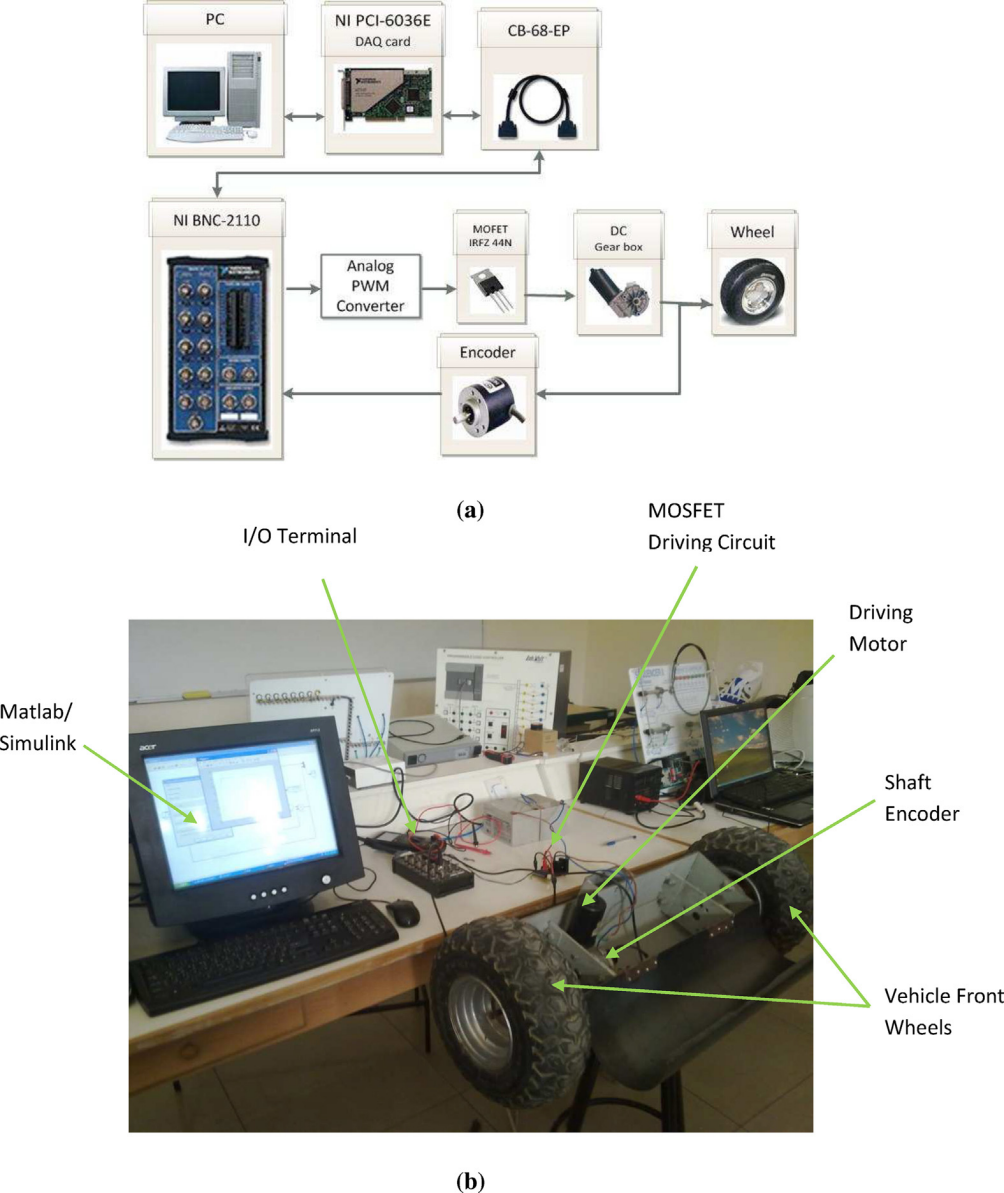
(a) Data acquisition system block diagram, (b) vehicle drive.

One of the driving motors was connected to the computer through a data acquisition system as shown in Fig. 8a while the lab experiment is shown in Fig. 8b. The advantage of applying system identification to the motor assembly is that the identified model represented the motor-bearing-wheel subsystem with all its parameters.

### Stage one: identification

**Fig. 9:**
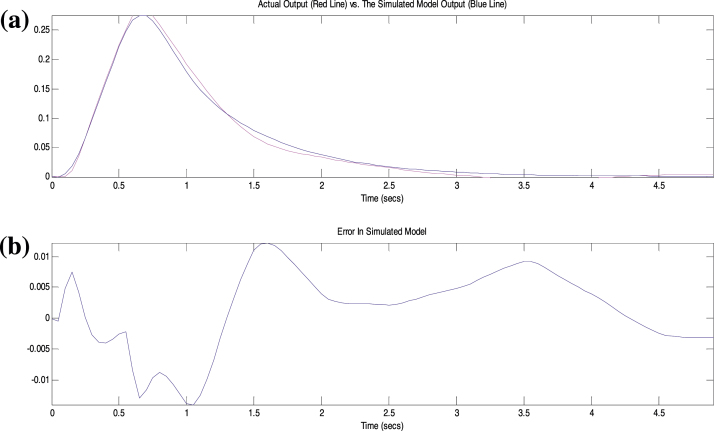
(a) Actual and identified model pulse speed response, (b) error between the actual and identified model responses.

The driving motor was identified online by applying an pulse signal to the real system and measuring its speed response. Fig. 9 shows the speed response of the real system versus the identified model. Several trials of different model orders were used and the 3rd order model showed the best match between the actual output and the predicted model output. The model transfer function was identified to be:

$$H(z)=\frac{0.0012262z}{z^{3}-2.3438z^{2}+1.8739z-0.52036}$$

### Stage two: controller design

**Fig. 10:**
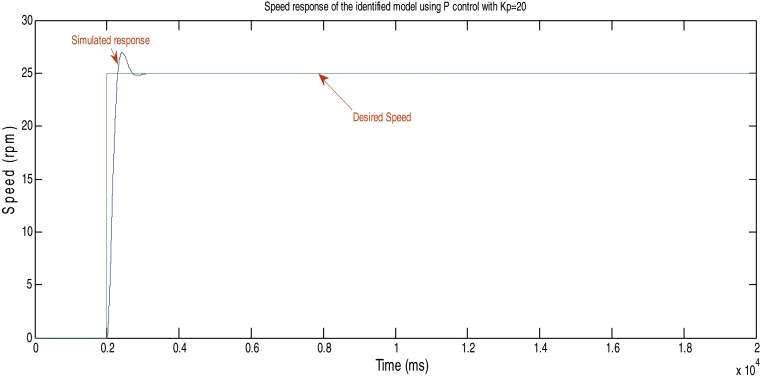
Simulated speed response of the system with off line control design.

**Fig. 11:**
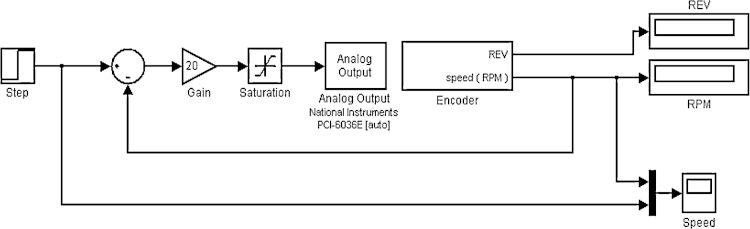
Simulink block diagram of online control.

The identified model was then used to design a proportional controller for the driving motor. The tuning objective was to get an overshoot less than 10% and settling time less than 0.2 s. Fig. 10 shows the speed response of the identified model where the system has an overshoot of 8% and a settling time of 0.1 s while the simulation blocks are shown in Fig. 11.

### Stage three: online control

**Fig. 12:**
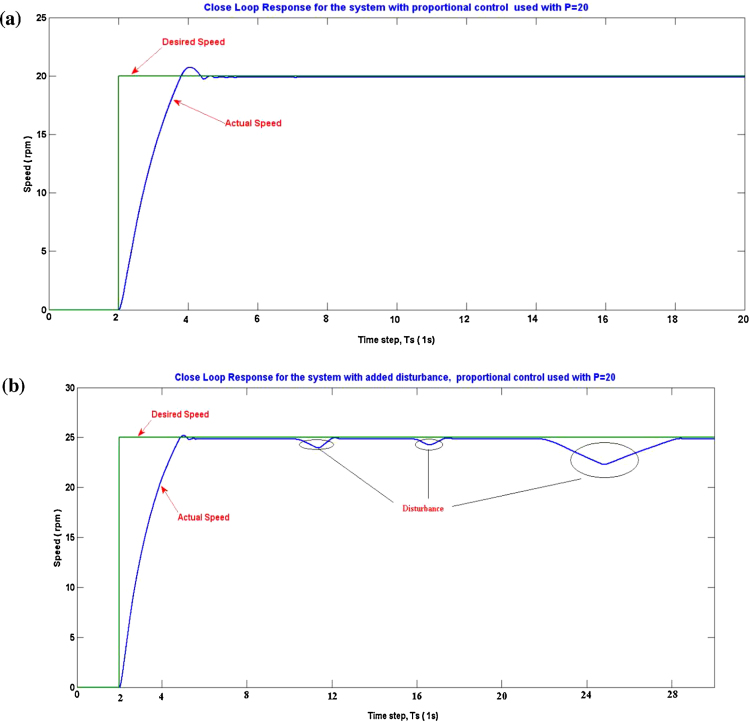
(a) Real system’s speed response, (b) real system’s speed response with added disturbance.

The designed controller (with the same tuned parameters) has been then applied to the real motor through the data acquisition system using the Simulink block diagram shown in Fig. 12. The speed response of the real motor is shown in Fig. 12a. Disturbances were introduced by holding the wheel during the steady state of the motor in order to test the ability of the controller to compensate for such errors. The controller was able to regulate the speed at 25 RPM as shown in Fig. 12b. The obtained results show the feasibility and reliability of the proposed method in practical applications. Moreover, it significantly reduced the time required for controller tuning and optimization.

## Additional information

Mechatronics is the synergistic integration of mechanical engineering with electronics and intelligent computer control for the design and manufacturing of products. Research activities cover a wide range of practical engineering issues which involve modelling, identification, and control of fully-integrated systems [5]. The design of mechatronic systems has been studied by many researchers. In [6], the mechatronic design process was described as a V-shape, a combination of top–down and bottom–up approaches, which included modelling, prototyping and integration. In [3], a mechatronic flow chart composed of several steps that included modelling and control design was presented. In [7], a description of hybrid simulation, design, and testing environments were presented. Those included Simulation-in-the-Loop (SIL), Hardware-in-the-Loop (HIL), and Control-Prototyping.

Topics in the area of identification and control of dynamic systems are of critical importance to mechatronic systems. Tutunji et al. [8] and Saleem et al. [4] used auto regressive moving average (ARMA) models and recursive least squares (RLS) algorithms to study and identify gyroscopic system behaviour and 3-phase induction motors, respectively. In [4], HIL environment was used for the identification and control of induction motors. HIL is a concept that combines virtual components with real system’s components and is mainly employed to test a real control system on a virtual plant in order to verify its performance before applying it to the real plant.

There is a vast amount of research in the areas of system identification, control, and HIL and therefore it would be very difficult to summarize all previous work. However, a variety of high-impact publications (from different journals and with different applications) were collected in order to provide a complete picture. The idea is to highlight the contribution of the proposed methodology.

System identification is an active research area that is closely related to control in mechatronics engineering. There are several books that have described the system identification area such as [9] and [10]. [9] is considered as a landmark in system identification methodology while [10] presented an update-to-date and comprehensive review of system identification methodology. The focus in these books was on system identification as a stand-alone research (i.e. without control design).

Other books, [1] and [11], incorporated system identification with control design. Landau and Zito showed how identified ARMA-based models can be used to design controllers while Liu described the use of Neural Networks for identification and control applications. Both books presented examples where real-data was collected in the labs to identify the plant and showed how the identified models can be used in simulation environment for the design of controllers (i.e. the first two stages in our proposed methodology).

In [12], researchers used frequency-based models to identify the dynamics of a dual-actuator hard disk drive system. The identified model was then used to improve the control tracking system. The controller was tested and implemented using a real-time DSP system, but they did not use a PC-based controller for testing the system. Therefore, duplication of such a setup requires knowledge in programming and implementing DSP-based controllers.

In [13], researchers developed multi-variable identification of a Boeing 747 in order to design a robust pitch/speed control. The final stage of their work was based on simulation runs only and therefore they did not implement their controller on the physical plant.

In [14], researchers used parameter identification methods to complete the nonlinear model of a micro helicopter. They also used the developed model to design a robust controller via simulation and applied the applied controller using dsPIC, but did not propose to use the PC as controller for final test of their system.

In [15], researchers used a combination of math models and identification methods to develop a twin rotor model. Also, they developed an observer and state-feedback controller using simulation and lab experiments. Even though they used a PC-based controller, the work involved advanced mathematical formulas and focused on the derivations rather than on a methodology that can be followed by others.

In [16], researchers worked on modeling and controlling a pancake DC limited angle torque motor. They used experimental data to identify parameters (such as friction and torque coefficients) and developed robust controllers. Their lab setup involved a real-time CompactRIO controller interfaced with a PC. Their emphasize was on the developed controller algorithms and the experimental results rather than on a system identification and control methodology that can be duplicated by others.

Researchers in the mechatronics field have used system identification as part of their design strategy where they incorporated HIL concept. Shetty and Kolk [3] presented a flow chart design strategy that included mathematical modeling, using physical laws or experimental data in the initial stages and HIL in the prototyping stage. The HIL simulation was described as *the process of fusing and synchronizing model, sensor, and actuator information is called real-time*. However, the design strategy did not include PC-based controller testing on the plant. Iserman [7] presented a mechatronics design strategy where he described two related concepts, HIL and control prototyping, where the HIL used real controller with simulated plant while the control prototyping used simulated controller with real plant. However, these concepts were not used as sequential parts of a controller design methodology and the use of the PC (as a controller) was not highlighted.

Many researchers worked on the HIL concept, but their methodology focused on developing real-time embedded controllers on virtual plants before applying the controller to the actual plants. This in turn requires the researcher to work with advanced applications and experimental setups.

In [17], researchers described an HIL simulation for the design and verification of an electro-hydraulic fuel control unit (FCU). In their work, the PC was used as to simulate the plant (integrated flight and engine dynamics) while the actual FCU was used. This differs from the third stage in our described methodology where the PC is used as the controller.

In [18], researchers integrated onboard hardware, flight control, ground station and software to realize the Hardware-in-the-Loop simulation in order to simulate and design appropriate flight controllers for UAV helicopter. They used the PC to simulate the helicopter flight dynamics and on-board hardware module to validate their work. In our case, we do the opposite where we use the real plant with PC-based controller.

In [19], researchers proposed a hybrid process simulation based on HIL concept where different components from manufacturing lines were replaced by simulated components. They showed the benefits of using HIL in manufacturing and developed a conceptual architecture that was applied to manufacturing cells. However, their work focused on testing manufacturing setups rather than developing controller algorithms.

In [20], researchers proposed a design methodology that incorporated HIL and rapid prototyping using embedded cRIO and Labview software platform for mobile vehicles. Their work is impressive, but highly complicated. Also, they did not use system identification methods in their work.

In [21], researchers developed a physics-based math model for a fuel-injection engine and used it as the simulated plant in developing real-time controllers using engine control modules. Their work did not involve experimental modeling and they did not use the PC as the controller.

In [22], researchers used system identification and control within HIL structure in order to design controllers for an actual-based mechanical system. They tested their controller using real-time dSpace controller board. Duplication of such a lab setup requires the availability of specific controllers and the knowledge in programming and implementing them.

In our work, an easy-to-follow three-stage methodology (based on PC and DAQ interface setup) is proposed, validated, and described. Therefore, the objective was to present a clear methodology that can be duplicated by others.

## Conclusions

In this paper, a methodology for the identification and control of electromechanical actuators was presented. In the proposed methodology, the plant-under-test was first identified by collecting real-time input/output data and running the identification algorithm online. Then, the controller was designed offline using simulation. Finally, the designed controller was applied to the plant in an HIL environment. The methodology is flexible and adaptive because it can incorporate a combination of different identification and control techniques. Furthermore, offline controller design avoids accidental damage to the plant-under-test and minimizes the design time. Two different electro-mechanical systems were used to validate the work. For each case, the plant was identified accurately and controlled properly where the identified model followed the plant’s dynamic behaviour with minimal error and the controller was able to meet the transient and steady-state specifications.

## Figures and Tables

**Table 1:** Induction motor specifications.

Variable	Rating
Nominal power	300 W
Nominal speed	2870 RPM
Nominal torque	1 N m
Nominal line voltage	220/380 V
Nominal frequency	50 Hz
Tachometer resolution	0.9 mV/RPM
